# Magnetic Healers and Sight Diagnosticians

**Published:** 1894-04

**Authors:** 


### MAGNETIC HEALERS AND SIGHT DIAGNOSTICIANS.

With the "boy phenomenon," who recently visited Austin and fleeced so many credulous people with his slight of hand performances, there was a "Professor"—some-sort-of-a-Hall, who "professed" to diagnose every case at sight. He would charge only a dollar for this non-essential,—for it seems that all was fish that came to their nets,—and turn the victim over to the tender mercies of the "phenomenon," who would then rub and manipulate with one hand while he pocketed a big fee with the other. The "phenomenon" was the owner of a "diploma," and had it registered; therefore he was not come-at-able; he was, under our laws, a "legal practitioner;" but the professor,—oh the professor, where was he at? Out in the cold, cold world of uncharity, without the saving clause of a diploma, and he got arrested. He was brought before Justice Barbour on a charge, preferred by Dr. Bennett, of practicing medicine without license or diploma. He pleaded that he was not practicing medicine; that he only made the diagnosis, and the "boy phenomenon," did the rest. The venerable Dr. Wooten was placed on the stand, and said that making a diagnosis, while not all of practicing medicine, was a large part of it; that any one who pretended to diagnose disease "at sight" (without examination) was a fraud, "it couldn't be did"; that not even small-pox could be so diagnosed.

The professor was bound over in $300 bond, to appear and stand trial at the next term of county court. But the professor skipped. It remains to be seen whether he will test the case, the probabilities being largely that he will find it cheaper to pay the $300 than to stand cost of trial; because, he would surely be convicted, and that would compel him either to go and get a diploma or get out of the State or quit "diagnosing," either of which would amount to more in the end than $300. He has too good a thing to give up; but if every town would stick him for $300 his revenue would be cut very materially. Let the profession look out for him, and follow Dr. Bennett's example.

It is a great pity there is not a law to reach the other fellow. He is heeled; he has a "diploma," more's the pity; but he takes fees,—and very large ones are paid him,—on promises to cure such cases as epilepsy and chronic arthritic rheumatism, cases which a physician would hold out no hopes of cure. It is a painful and unpleasant thing for a physician to do as Dr. Bennett did in Hall's case. He lays himself liable to be accused of jealousy, of meddlesomeness, etc.; he is not given credit for honesty and disinterestedness. And still, if a physician does not bring the charge and make an effort to expose these traveling quacks, no one will, and they, unmolested, reap a rich harvest, carrying away vast sums from every town, taken from the credulous afflicted, who will grasp at a straw. That is a singular phase in human nature which makes an incurable cling to hope, and believe readily the promise of a stranger to cure him, even after his old family physician, a man grown gray, may be, in the honorable practice of medicine, has told him he could not be cured. It will be claimed, however, that the "boy phenomenon" did effect cures. Where are they? It remains to be seen. Under his ministrations some seemed to get better; but will the relief, if relief, be more than temporary? For awhile the will power is stimulated, an afflicted one will "make an effort,"—his hope is stimulated; he has faith, his imagination comes into play, and for the time being all these work together to produce apparent improvement. Just so they would do under the ministrations of any one who could command the confidence—as these traveling fellows seem to have the strange power of doing.

Well, experience is a dear teacher, and these deluded people who gave up their hard earned money to those two on promise to cure an afflicted child of epilepsy, perhaps, will be wiser men, may be, if poorer.

